# A missense variant of the *ABCC11* gene is associated with Axillary Osmidrosis susceptibility and clinical phenotypes in the Chinese Han Population

**DOI:** 10.1038/srep46335

**Published:** 2017-05-09

**Authors:** Yunqing Ren, Wenting Liu, Jisu Chen, Jianyou Wang, Ke Wang, Jiong Zhou, Suiqing Cai, Min Zheng, Jianjun Liu, Lunfei Liu, Dan Xue

**Affiliations:** 1 Department of Dermatology, Second Affiliated Hospital, Zhejiang University School of Medicine, Hangzhou, Zhejiang, China; 2 Human Genetics, Genome Institute of Singapore, Singapore; 3 Department of Oncology, Second Affiliated Hospital, Zhejiang University School of Medicine, Hangzhou, Zhejiang, China; 4 Department of Dermatology, Fourth Affiliated Hospital, Zhejiang University School of Medicine, Hangzhou, Zhejiang, China; 5 Department of Plastic Surgery, Second Affiliated Hospital, Zhejiang University School of Medicine, Hangzhou, Zhejiang, China

## Abstract

Axillary osmidrosis (AO) is a common condition characterized by an offensive odor arising from apocrine gland secretions in the axillae that socially and psychologically impairs affected individuals. The exact aetiology of AO is still not fully understood, but genetic factors have been suggested to play an important role. Recently, a single nucleotide polymorphism (SNP) rs17822931 in the *ABCC11* gene located on human chromosome 16q12.1 has been shown to be associated with AO. In this study, we genotyped rs17822931 in two independent samples of Chinese Hans including 93 AO individuals vs 95 controls and 81 AO individuals vs 106 controls by using SNaPshot Multiplex Kit. We confirmed the association for *ABCC11* gene, showing that rs17822931-G was significantly associated with increased risk for AO (P_combined_ = 1.42E-21, OR = 83.94, 95% CI = 83.03–84.85). We also found rs17822931 was associated with subphenotypes of AO. AO individuals carrying the risk allele G are more likely to show wet earwax (P = 2.40E-05), higher frequency of family history (P = 1.04E-02) and early age of onset (P = 3.81E-02). Our study concluded that the association of rs17822931 in the *ABCC11* gene with AO was replicated in Chinese Han population.

Axillary Osmidrosis (AO) is known as a chronic skin condition characterized by strong body odor from the armpits resulting from excessive secretions of apocrine glands in the axilla that are converted to odoriferous compounds by bacteria. The symptoms of AO generally develop around puberty and tend to be more prevalent in people of African descent as well as European populations^1^. It does not affect physical health, but can be annoying and distressing, and can cause considerable personal and social embarrassment to persons, especially in Asian countries where individuals with strong body odor comprise a minor population^2^.

It has been evident that axillary odor is due to the biotransformation of odourless secretions into volatile odorous molecules by cutaneous microorganisms. However, the exact mechanism of AO is complex and still not fully understood. Interestingly, the effect of genetic factors has also been suggested to play an important role in the development of AO^3^. It has been reported about 94% of AO cases have family history, and some showed autosomal dominant pattern of inheritance^4^. Recently, strong evidence has discovered showing that a nonsynonymous coding variant rs17822931(538G > A) in exon 4 of *ABCC11* gene, which was originally identified as a genetic determinant of human earwax type^5^, is strongly associated with AO development^6^,^7^. *ABCC11*, also known as multidrug resistance associated protein 8 (*MRP8*), is a member of the human ABC transporter gene family. It is expressed in apocrine glands, and may have a key function in the secretion of odorants and their precursors^8^,^9^. Subsequent studies revealed that individuals carrying rs17822931-G allele have a very high risk for developing AO in multiple populations^6^,^7^,^8^. However, the association of rs17822931 with AO remains to be confirmed in Chinese population. Rs17822931-G was first shown to co-segregate with the AO phenotype in a single Chinese pedigree with 9 AO individuals^10^. In a subsequent study^11^, rs17822931 was genotyped in 40 AO cases and 5 normal controls, but the association of rs17822931 could not be properly evaluated due to the insufficient number of controls analyzed.

In the current study, we tested the association of rs17822931 in a large Chinese AO cohort consisting of two independent samples. Our study has not only confirmed the previously reported association with robust evidence, but also revealed the association of rs17822931 with the clinical phenotypes of AO in Chinese population.

## Results

### Characteristics of Study Subjects

Two independent samples, consisting of a total of 174 AO individuals (range from 17 to 64 years) and 201 controls (range from 18 to 88 years), were investigated. The distribution of cases and controls according to age, gender and the characteristics of earwax type, age of onset and family history information for the cases were summarized in Table 1. The cases (average age = 25.82 ± 7.79 years) were younger than the controls (average age = 43.33 ± 15.54 years), and a moderate difference in gender ratio was also observed between the cases (62.1% being females) and controls (53.2% being females). Positive family history was observed in 77.59% (135/174) of the AO individuals. 91.38% (159/174) of the AO individuals had wet-type earwax, which is consistent with the reported association between axillary odor and the wet-type earwax.

### Association of AO with SNP rs17822931

The results of the association analysis of rs17822931 in the two independent samples were summarized in Table 2. The genotypes of rs17822931 was in Hardy–Weinberg equilibrium in the controls (P_control_ = 0.42 in all the controls). Because the gender and age were not fully matched between the cases and controls, all the association analyses were performed by adjustment for gender and age. We found consistent significant association of this SNP with AO between the sample 1 (P = 2.49E-14, OR = 59.74, 95% CI = 20.87–171) and sample 2 (P = 4.20E-09, OR = 231.4, 95% CI = 37.65–1422) without any evidence for heterogeneity. Then we performed the meta-analysis of the combined two independent samples. Rs17822931-G showed highly significant association in the combined samples of 174 cases and 201 controls (P = 1.42E-21, OR = 83.94, 95% CI = 83.03–84.85). In addition, we also evaluated the performance of risk prediction by the SNP rs17822931, and the AUC value is 0.918.

### Stratified association analysis by clinical subtypes

We also performed the association analysis of the SNP rs17822931 by stratifying AO individuals into clinical subtypes according to earwax type, age of onset and family history. The stratified association results were summarized in Table 3. Rs17822931-G showed significantly stronger risk effect in cases with wet earwax than the ones with dry earwax (OR = 143.80 vs 7.58, P = 2.40E-05), cases with positive family history than the ones without family history (OR = 195.10 vs 18.14, P = 1.04E-02), and cases with early-onset AO (≤14 years) than the ones with late-onset AO (OR = 123.40 vs 55.26, P = 3.81E-02).

## Discussion

In this study, we have confirmed the previously reported association between AO and rs17822931 in *ABCC11* with robust and conclusive evidence in Chinese population. In addition, our study has also shown the association of rs17822931 with earwax type, family history and age of onset. While the association with earwax type in AO individuals is well expected, the current study is the first to demonstrate the genetic effect of rs17822931 in the AO individuals on positive family history and early onset-set of AO.

Rs17822931 in the *ABCC11* gene was first demonstrated to be the genetic determinant of human earwax type^5^, which has been viewed as a landmark discovery, representing the first example of DNA polymorphism determining a visible phenotypic trait. Given the long recognized strong association of AO with wet earwax, the association of rs17822931 with AO was suspected and later confirmed by multiple studies. And subsequent molecular studies has further demonstrated the strong impact of this SNP on the protein expression level and function of *ABCC11*^7^,^8^,^12^. It has been showed that *ABCC11* encodes an protein component of apical efflux pump and plays an important role in the secretion of key body odorants ((E)-3-methyl-2-hexenoic acid (E-3M2H), 3-methyl-3-sulfanylhexanol (3M3SH) and 3-hydroxy-3-methyl-hexanoic acid(HMHA)) and their precursors (the amino-acid conjugates 3M2H–Gln, HMHA—Gln, and Cys–Gly–(S) 3M3SH) from apocrine sweat glands^8^,^13^. While the GG and GA genotypes of this SNP were found to be associated with high levels of the odorants and their amino acid conjugates in axillary extracts, the AA genotype was found to be associated with a near-complete loss of these malodorants and precursors. Rodriguez *et al*. analyzed the association between rs17822931 and deodorant usage from a large population (~17000 individuals) and showed that AA genotypes were almost 5-fold overrepresented in individuals who never use deodorant or use it infrequently^14^. Recently, Prigge *et al*.^15^ examined axillary odorants among 30 male individuals of African-American, Caucasian and East Asian with respect to their *ABCC11* genotype, and confirmed that they all produce measurable amounts of the same axillary odorants but East Asians with TT homozygotes (described as AA genotype in current study) produce significantly less of the characteristic axillary odorants, suggesting that key characteristic axillary odorants do not strictly vary with *ABCC11* genotype. It has also been suggested that the sources that contribute to axillary odor are diverse and complex^16^,^17^, and previous studies have identified several other proteins, such as Apolipoprotein D (*ApoD*)^18^ and γ-glutamyl-transferase (*GGT1*)^13^, that are involved in transportation and metabolic processes of characteristic axillary odorants. Taking together, these findings suggested that there are other biochemical pathways (beyond *ABCC11*) involved in the regulation of transport and/or release of volatile odorants in the axilla.

Interestingly, the genotypes of rs17822931 showed a strong deviation from HWE in the AO cases with most of the cases to be heterozygous. The allele frequency of rs17822931 observed in our samples is consistent with the previously published frequency in Chinese population (Supplementary Table S1). Giving that our cases and control samples were genotyped together, and the genotypes of the SNP in the controls did not show any evidence of deviation from HWE (P_control_ = 0.42), the excessive heterozygosity observed in the cases is unlikely due to poor genotyping analysis. In addition, the similar excessive heterozygosity of the SNP in the AO cases has also been observed in previous studies (Supplementary Table S2) and is therefore biological. It has been long recognized that this variant shows tremendous frequency diversity across world populations and has been suggested to be under strong selection^19^,^20^. From the HapMap and ALFRED (The allele frequency database) data, while G allele shows higher frequency in European and African (70–100%) populations, the A allele is more frequent in East Asians (70–100%), particularly in China and Korea. The excessive heterozygosity observed in AO individuals is probably due to the effect of selection, particularly nonrandom mating against AO phenotype. However, the mechanism remains to be elucidated.

In summary, our study has provided conclusive evidence for the association of rs17822931 in the *ABCC11* with the susceptibility of AO in the Chinese Han population. In addition, our study has also demonstrated the association of rs17822931 with the clinical phenotypes of AO. Our findings have advanced understanding on the genetic predisposition to AO, although further studies are needed to show molecular mechanism for this functional variant to cause the development of AO.

## Methods

### Study Subjects

All subjects were collected from the Department of plastic Surgery and Department of Dermatology at Second Affiliated Hospital of Zhejiang University School of Medicine at Hangzhou, Zhejiang, China. All cases and controls were unrelated individuals of Han Chinese ethnicity by self-report. The diagnosis of AO was made on presenting typical symptoms of axillary odor and receiving surgery in the clinics to remove axillary apocrine glands. Clinical information including age, gender, age of onset, family history, earwax type was recorded from the cases through a full medical check-up. A total of 201 controls without AO were recruited from health examination center simultaneously. All the participants were collected with written informed consent, and the study protocol was approved by the Ethics Committee of Zhejiang University School of Medicine and was conducted according to the Declaration of Helsinki Principles.

### DNA isolation and genotyping

Genomic DNA of each sample was extracted from peripheral blood leukocytes using FlexiGene DNA kits (Qiagen, Germany). SNP rs17822931 was genotyped by SnaPshot Multiplex Kit (Applied Biosystems Co., USA). All procedures were performed according to the manufacturer’s instructions. The primers used were as follows:rs17822931_Forward:ACGTTGGATGACAAGGTTGATTTTCGATGC, rs17822931_Reverse: ACGTTGGATGAGTTCCATCGCTAAACCTCT.

### Statistical analyses

Association analysis was performed by using logistic regression additive model with gender and age as covariates in PLINK v1.07^21^. The joint analysis of the two independent samples was performed using an inverse variance meta-analysis assuming fixed-effects, with a Cochran’s Q test and I^2^ to assess between-study heterogeneity. Hardy-Weinberg proportion was tested in control samples to ensure genotyping quality and passed the test with P values > 0.05. The association of clinical phenotypic traits was analyzed by comparing cases with/without a certain sub-phenotype with controls using logistic additive regression model with gender and age as covariates. We also performed case-only analysis to examine the significance of the differential risk effects of the SNP on the subtypes of AO.

To evaluate the power of SNP for predicting susceptibility risk, we calculate the AUC (area under the ROC curve) value of prediction for the SNP by using additive model with age and gender as covariates. Specifically, the prediction of risk allele is computed from the multiplication of beta = ln(OR) score from the prediction logistic model with the number of risk alleles in each sample. The AUC score is computed from R package “ROCR”.

## Additional Information

**How to cite this article:** Ren, Y. *et al*. A missense variant of the *ABCC11* gene is associated with Axillary Osmidrosis susceptibility and clinical phenotypes in the Chinese Han Population. *Sci. Rep.* **7**, 46335; doi: 10.1038/srep46335 (2017).

**Publisher's note:** Springer Nature remains neutral with regard to jurisdictional claims in published maps and institutional affiliations.

## Supplementary Material

Supplementary Table

## Figures and Tables

**Table 1:** Characteristics of the AO Cases and Controls in this study.

Analysis sample	Cases						Controls		
	Sample size	Male/Female	Mean age(s.d.)	Mean age of onset (s.d.)	Familial/Sporadic	Wet/Dry earwax	Sample size	Male/Female	Mean age(s.d.)
Sample 1	93	36/57	28.05 (8.45)	16.24(3.17)	66/27	83/10	95	55/40	41.47(13.46)
Sample 2	81	30/51	23.25 (6.06)	14.56 (2.31)	69/12	76/5	106	39/67	45.00(17.08)
Total	174	66/108	25.82 (7.79)	15.45 (2.92)	135/39	159/15	201	94/107	43.33(15.54)

**Table 2:** Summary of the association results for rs17822931 in two independent samples as well as the combined sample.

	Sample 1				Sample 2				Combined					
	Allele (Frequency)		OR (95% CI)	P^a^	Allele (Frequency)		OR (95% CI)	P^a^	Allele (Frequency)		OR (95% CI)	P^b^	Q^c^	I^d^
	G	A			G	A			G	A				
Case	86 (0.46)	100 (0.54)	59.74 (20.87–171)	2.49E-14	82 (0.51)	80 (0.49)	231.4 (37.65–1422)	4.20E-09	168 (0.48)	180 (0.52)	83.94 (83.03–84.85)	1.42E-21	0.21	37.49
Control	8 (0.04)	182 (0.96)			13 (0.06)	199 (0.94)			21 (0.05)	381 (0.95)				

^a^p value for the risk allele from the logistic regression with gender and age as covariates. ^b^P value from the meta analysis. ^c^p value for Cochrane’s Q statistic, ^d^I^2^ heterogeneity index.

**Table 3:** Stratified association analysis of rs17822931 by clinical subgroups.

Samples	Total	Genotype			OR	95% CI	P
		GG	GA	AA			
**Earwax type**							
Wet	159	5	149	5	143.80	(51.61–400.40)	2.40E-05
Dry	15	0	9	6	7.58	(2.54–22.63)	
**Family history**							
Familial	135	3	129	3	195.10	(58.84–646.60)	1.04E-02
Sporadic	39	2	29	8	18.14	(7.22–45.60)	
**Age of onset**							
≤14 years	80	5	72	3	123.40	(32.10–474.30)	3.81E-02
>14 years	94	0	86	8	55.26	(23.07–132.30)	

OR was calculated between subgroups and controls by the logistic regression with gender and age as covariates, P value was from the comparison between the clinical subgroups by the logistic regression.
