# Supplementary material for: A missense variant of the ABCC11 gene is associated with Axillary Osmidrosis susceptibility and clinical phenotypes in the Chinese Han Population
Source: Sci Rep. 2017 May 9;7:46335. doi: 10.1038/srep46335 (PMC5423033; doi:10.1038/srep46335)
Supplement: Supplementary Table [file srep46335-s1.doc]

**A missense variant of the *ABCC11* gene is associated with Axillary Osmidrosis susceptibility and clinical phenotypes in the Chinese Han Population**

Yunqing Ren^1^, Wenting Liu^2^, Jisu Chen^1^, Jianyou Wang^1^, Ke Wang^3^, Jiong Zhou^1^ Suiqing Cai^1^, Min Zheng^1^, Jianjun Liu^2^, Lunfei Liu^1,4^*& Dan Xue^5^*.

^1^Department of Dermatology, Second Affiliated Hospital, Zhejiang University School of Medicine, Hangzhou, Zhejiang, China.

^2^Human Genetics, Genome Institute of Singapore, Singapore.

3Department of Oncology, Second Affiliated Hospital, Zhejiang University School of Medicine, Hangzhou, Zhejiang, China.

^4^Department of Dermatology, Fourth Affiliated Hospital, Zhejiang University School of Medicine, Hangzhou, Zhejiang, China.

^5^Department of Plastic Surgery, Second Affiliated Hospital, Zhejiang University School of Medicine, Hangzhou, Zhejiang, China.

*Correspondence and requests for materials should be addressed to D.X. (xuedan@zju.edu.cn) or L.F.L(liulunfei@medmail.com.cn)

**Supplementary Table 1**.  Genotypes and frequencies of rs17822931 of ABCC11 among different ethinc populations in previous study.

| Ethnic populations | AA | GA | GG | No. of individuals genotyped | Frequency of allele A |
| --- | --- | --- | --- | --- | --- |
| Korean | 99 | 0 | 0 | 99 | 1 |
| Chinese | 475 | 82 | 8 | 565 | 0.913 |
| Mongolian | 126 | 36 | 4 | 166 | 0.867 |
| Japanese | 162 | 99 | 11 | 272 | 0.778 |
| Vietnamese | 82 | 60 | 11 | 153 | 0.732 |
| Dravidian | 27 | 17 | 6 | 50 | 0.71 |
| Thai | 248 | 196 | 108 | 552 | 0.627 |
| Vedda | 7 | 12 | 1 | 20 | 0.65 |
| Indonesian | 66 | 115 | 103 | 284 | 0.435 |
| Malaysian | 32 | 67 | 33 | 132 | 0.496 |
| Taiwanese | 34 | 48 | 21 | 103 | 0.563 |
| Native American | 6 | 8 | 6 | 20 | 0.5 |
| Philippino | 11 | 23 | 14 | 48 | 0.469 |
| Easter Islander | 4 | 18 | 7 | 29 | 0.448 |
| Bolivian | 5 | 14 | 11 | 30 | 0.4 |
| Kazakh | 6 | 11 | 13 | 30 | 0.383 |
| Native Paraguayan | 2 | 48 | 75 | 125 | 0.208 |
| Russian | 5 | 45 | 62 | 112 | 0.246 |
| Solomon Islander | 2 | 25 | 35 | 62 | 0.234 |
| Pacific islander | 1 | 1 | 5 | 7 | 0.214 |
| French | 1 | 3 | 8 | 12 | 0.208 |
| Andean people | 1 | 2 | 7 | 10 | 0.2 |
| Hungarian | 0 | 4 | 6 | 10 | 0.2 |
| Jewish | 0 | 4 | 6 | 10 | 0.2 |
| Ukrainian | 0 | 15 | 27 | 42 | 0.179 |
| Papuan | 1 | 11 | 26 | 38 | 0.171 |
| European American | 1 | 16 | 65 | 82 | 0.11 |
| Vanuatu islander | 1 | 17 | 74 | 92 | 0.103 |
| Iberian | 0 | 2 | 8 | 10 | 0.1 |
| Colombian | 0 | 2 | 15 | 17 | 0.059 |
| Venezuelan | 0 | 3 | 29 | 32 | 0.047 |
| African | 0 | 1 | 10 | 11 | 0.045 |
| African American | 0 | 0 | 10 | 10 | 0 |
| Our study (controls) | 181 | 19 | 1 | 201 | 0.947 |

Data are from Yoshiura et al.(2006)[^1^](#_ENREF_1) and our study.

**Supplementary Table 2**. Genotypes and frequencies of rs17822931 of ABCC11 in AO individuals.

|  |  |  |  |  | Genotype at the rs17822931 locus | | |  | Allele frequency | | Earwax type | |
| --- | --- | --- | --- | --- | --- | --- | --- | --- | --- | --- | --- | --- |
| Subject studied | |  | total |  | GG | GA | AA |  | A | G | Wet | Dry |
| Individuals with AO (Kyushu)^a^ | | | 79 |  | 5 | 73 | 1 |  | 0.4746835 | 0.5253165 | 78 | 1 |
| Individuals with AO (Okinawa) ^a^ | | | 38 |  | 3 | 34 | 1 |  | 0.4736842 | 0.5263158 | 37 | 1 |
| Individuals with AO (Nagasai) ^a^ | | | 41 |  | 2 | 39 | 0 |  | 0.4756098 | 0.5243902 | 41 | 0 |
| Individuals with AO (Chinese) ^b^ | | | 40 |  | 3 | 37 | 0 |  | 0.462 | 0.5375 | NA | NA |
| Individuals with AO (Chinese) ^c^ | | | 174 |  | 5 | 158 | 11 |  | 0.5172414 | 0.4827586 | 159 | 15 |

Data are calculated from ^a^Nakano et al.(2009)[^2^](#_ENREF_2), ^b^ Sun et al.(2013)[^3^](#_ENREF_3) and ^c^ this paper.

**References**

1 Yoshiura, K. *et al.* A SNP in the ABCC11 gene is the determinant of human earwax type. *Nat Genet* **38**, 324-330 (2006).

2 Nakano, M., Miwa, N., Hirano, A., Yoshiura, K. & Niikawa, N. A strong association of axillary osmidrosis with the wet earwax type determined by genotyping of the ABCC11 gene. *BMC Genet* **10**, 42 (2009).

3 Sun, Y., Long, J. & Wang, Y. Correlation between ABCC11 gene single nucleotide polymorphism and the incidence of axillary osmidrosis in Chinese Han population. *Zhong Nan Da Xue Xue Bao Yi Xue Ban* **38**, 1141-1145 (2013).
